# Diagnostic Performance Assessment of Saliva RT-PCR and Nasopharyngeal Antigen for the Detection of SARS-CoV-2 in Peru

**DOI:** 10.1128/spectrum.00861-22

**Published:** 2022-07-18

**Authors:** Roger I. Calderón, Tulip A. Jhaveri, Marco A. Tovar, J. Santiago Palomino, Nadia N. Barreda, Oswaldo M. Sanabria, Jesús Peinado, Claudio Ramirez, L. Fernando Llanos Zavalaga, Gissela Valderrama, Molly F. Franke, Carole D. Mitnick, Leonid Lecca, Gustavo E. Velásquez

**Affiliations:** a Socios En Salud Sucursal Peru, Lima, Peru; b Faculdade de Medicina, Universidade Federal do Rio de Janeiro, Rio de Janeiro, Brazil; c Grupo de Investigación en Bioquímica y Biología Sintética, Universidad Nacional Federico Villarreal, Lima, Peru; d Division of Infectious Diseases, Department of Medicine, University of Mississippi Medical Center, Jackson, Mississippi, USA; e Division of Medical Microbiology, Department of Pathology, Brigham and Women’s Hospital, Harvard Medical School, Boston, Massachusetts, USA; f Escuela de Medicina, Facultad de Ciencias de la Salud, Universidad Peruana de Ciencias Aplicadas, Lima, Peru; g Dirección de Redes Integradas de Salud Lima Norte, Lima, Peru; h Department of Global Health and Social Medicine, Harvard Medical School, Boston, Massachusetts, USA; i Division of Global Health Equity, Brigham and Women’s Hospital, Boston, Massachusetts, USA; j Partners In Health, Boston, Massachusetts, USA; k UCSF Center for Tuberculosis, University of California, San Francisco, California, USA; l Division of HIV, Infectious Diseases, and Global Medicine, University of California, San Francisco, California, USA; Johns Hopkins Hospital

**Keywords:** antigen, COVID-19, diagnosis, nasopharyngeal swab, Peru, RT-PCR, saliva, SARS-CoV-2, validation

## Abstract

Widely available and reliable testing for SARS-CoV-2 is essential for the public health response to the COVID-19 pandemic. We estimated the diagnostic performance of reverse transcription PCR (RT-PCR) performed on saliva and the SD Biosensor STANDARD Q antigen test performed on nasopharyngeal swab compared to the reference standard, nasopharyngeal swab (NP) RT-PCR. We enrolled participants living and/or seeking care in health facilities in North Lima, Peru from November 2020 to January 2021. Consenting participants underwent same-day RT-PCR on both saliva and nasopharyngeal swab specimens, antigen testing on a nasopharyngeal swab specimen, pulse oximetry, and standardized symptom assessment. We calculated sensitivity, specificity, and predictive values for the nasopharyngeal antigen and saliva RT-PCR compared to nasopharyngeal RT-PCR. Of 896 participants analyzed, 567 (63.3%) had acute signs/symptoms of COVID-19. The overall sensitivity and specificity of saliva RT-PCR were 85.8% and 98.1%, respectively. Among participants with and without acute signs/symptoms of COVID-19, saliva sensitivity was 87.3% and 37.5%, respectively. Saliva sensitivity was 97.4% and 56.0% among participants with cycle threshold (*C_T_*) values of ≤30 and >30 on nasopharyngeal RT-PCR, respectively. The overall sensitivity and specificity of nasopharyngeal antigen were 73.2% and 99.4%, respectively. The sensitivity of the nasopharyngeal antigen test was 75.1% and 12.5% among participants with and without acute signs/symptoms of COVID-19, and 91.2% and 26.7% among participants with *C_T_* values of ≤30 and >30 on nasopharyngeal RT-PCR, respectively. Saliva RT-PCR achieved the WHO-recommended threshold of >80% for sensitivity for the detection of SARS-CoV-2, while the SD Biosensor nasopharyngeal antigen test did not.

**IMPORTANCE** In this diagnostic validation study of 896 participants in Peru, saliva reverse transcription PCR (RT-PCR) had >80% sensitivity for the detection of SARS-CoV-2 among all-comers and symptomatic individuals, while the SD Biosensor STANDARD Q antigen test performed on nasopharyngeal swab had <80% sensitivity, except for participants whose same-day nasopharyngeal RT-PCR results showed cycle threshold values of <30, consistent with a high viral load in the nasopharynx. The specificity was high for both tests. Our results demonstrate that saliva sampling could serve as an alternative noninvasive technique for RT-PCR diagnosis of SARS-CoV-2. The role of nasopharyngeal antigen testing is more limited; when community transmission is low, it may be used for mass screenings among asymptomatic individuals with high testing frequency. Among symptomatic individuals, the nasopharyngeal antigen test may be relied upon for 4 to 8 days after symptom onset, or in those likely to have high viral load, whereupon it showed >80% sensitivity.

## INTRODUCTION

The coronavirus disease 2019 (COVID-19) pandemic, caused by severe acute respiratory syndrome coronavirus 2 (SARS-CoV-2), has led to an unprecedented global crisis. Countries with under-resourced health systems are particularly affected (1, 2). In mid-2021, Peru reported 5,551 COVID-19 deaths per one million people, the highest mortality rate worldwide (3). Surveillance and response have relied on identification, isolation, and monitoring of persons with COVID-19; contact tracing; and quarantining exposed individuals to interrupt transmission (4). Widely available, easily implementable, and accurate testing for SARS-CoV-2 is essential to reduce transmission. Nasopharyngeal swab (NP) reverse transcription PCR (RT-PCR) has been used routinely for diagnosis of SARS-CoV-2 (5). However, reliance on RT-PCR testing using NP swabs has drawbacks, namely: risks to the health personnel collecting samples, limited reagent supply, limited capacity for and high cost of performing RT-PCR, and the discomfort of sample collection. Options for improving the experience and availability of testing include the use of alternatives to NP samples, rapid tests, and/or self-testing. Here, we investigate the first two of these options.

Saliva, an alternative and less invasive sample than NP swabs, has previously been used for diagnosis of other infections (6, 7); the possibility of unsupervised collection minimizes health personnel exposure. Several studies have demonstrated its potential as a specimen type for SARS-CoV-2 detection (8, 9). Another alternative is rapid testing: in mid-2020, the World Health Organization (WHO) recommended two SARS-CoV-2 antigen detection tests: Panbio COVID-19 Ag Rapid Test Device (Abbott Rapid Diagnostics Jena GmbH, Germany) and STANDARD Q COVID-19 Ag Test (SD Biosensor, Inc., Republic of Korea) (10, 11). The diagnostic performance of these tests has varied across settings (12). Nevertheless, both have marketing authorization in Peru without having been evaluated there. The absence of local validation has hindered routine uptake of these tests, which could be important, affordable tools in the COVID-19 response.

The combination of alternatives to NP swabs collected by health care workers and antigen tests creates the potential for rapid, cost-effective diagnostic testing that can assist with faster diagnosis, thereby preventing further spread of the virus. This report compares the performance characteristics of saliva-based RT-PCR and NP-based rapid antigen testing against the standard of care NP RT-PCR test. We compared the sensitivity of each test in the whole study population, and in subgroups classified by presence or absence of symptoms and by cycle threshold (*C_T_*) threshold values, to the 80% threshold recommended by the WHO.

## RESULTS

**FIG 1:**
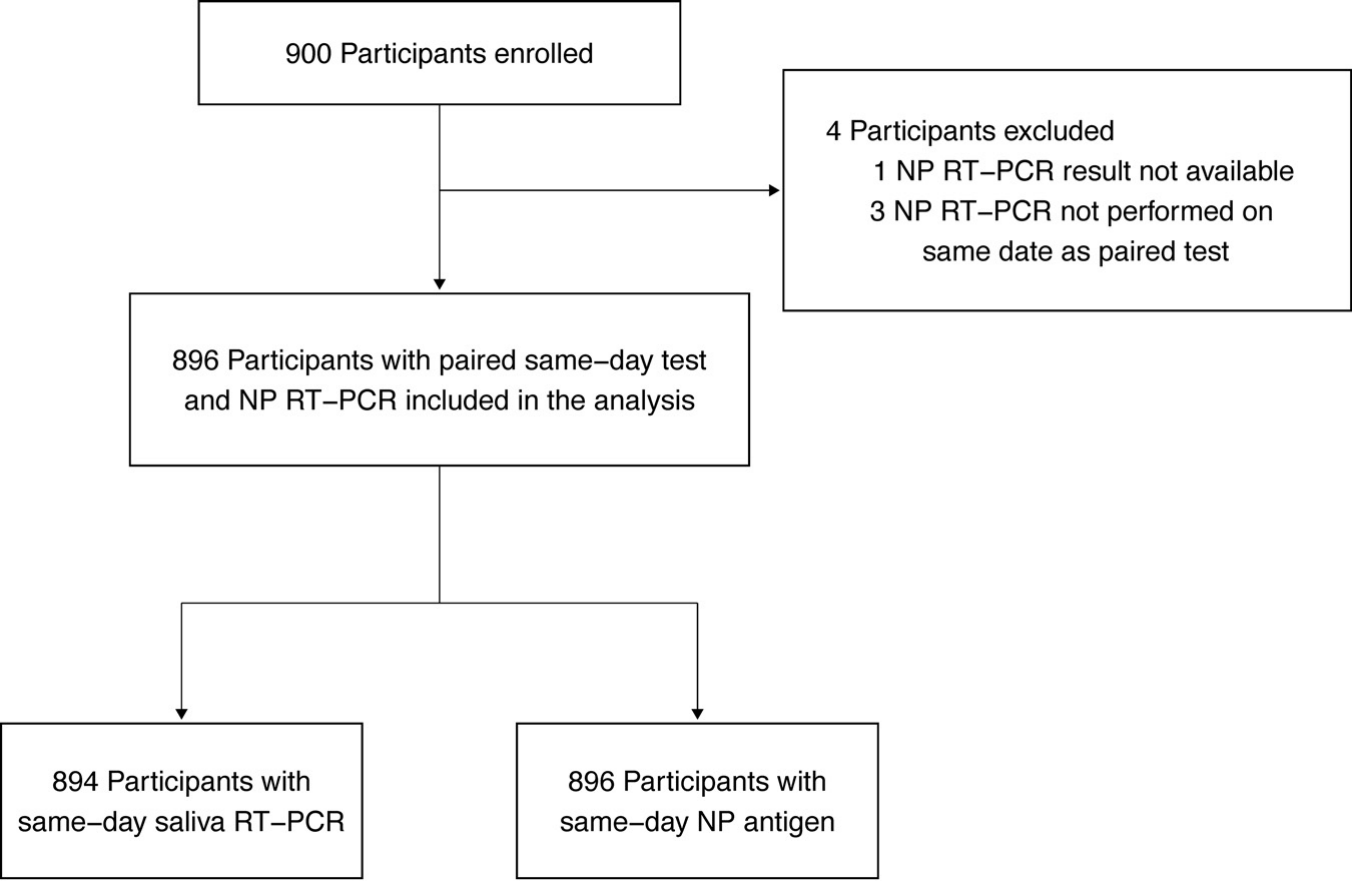
Flow diagram of participants included in the validation study.

Among 900 enrolled participants, 896 who provided an NP sample for RT-PCR were included in the analysis (Fig. 1). Of these, 894 had a same-day saliva sample for RT-PCR and 896 had a same-day NP sample for antigen testing. All participants were unvaccinated. Baseline demographic and clinical characteristics are shown in Table 1. Median participant age was 40 years, 562 (62.7%) were female, and 256 (28.6%) had comorbidities. Within 30 days prior to the enrollment visit, 322 (35.9%) reported exposure to SARS-CoV-2 and 567 (63.3%) had onset of acute signs/symptoms of COVID-19. Symptomatic participants underwent testing within a median 3 (interquartile range [IQR], 5 to 8) days from onset of acute signs/symptoms of COVID-19 and reported a median of 4 (IQR, 3 to 5) symptoms; the most frequently reported symptoms included cough, fever, headache, malaise, and sore throat. Of 896 participants tested, 269 (30.0%) tested positive by NP RT-PCR, 242/894 (27.1%) tested positive by saliva RT-PCR, and 201 (22.4%) tested positive by NP antigen (Table 2).

**TABLE 1:** Characteristics of participants tested for SARS-CoV-2 infection^*a*^

Characteristic	Total (*N* = 896)
Median age in yrs (IQR)	40 (28–54)
Sex	
Female	562 (62.7%)
Male	334 (37.3%)
Previous test for SARS-CoV-2	
No previous test	680 (75.9%)
RT-PCR negative	9 (1.0%)
Rapid antibody negative	167 (18.6%)
RT-PCR positive	3 (0.3%)
Rapid antibody positive	37 (4.1%)
Contact with presumptive or known COVID-19 case in the past 4 wks	
Yes	322 (35.9%)
No	574 (64.1%)
Comorbidity	
Yes	256 (28.6%)
No	640 (71.4%)
Type of comorbidity^*b*^	
Cardiovascular disease, including hypertension	84 (9.4%)
Chronic pulmonary disease	5 (0.6%)
Diabetes	47 (5.2%)
Immunodeficiency, including HIV infection	4 (0.4%)
Obesity	42 (4.7%)
Pulmonary tuberculosis	3 (0.3%)
Other	121 (13.5%)
Acute signs/symptoms of COVID-19	
Yes	567 (63.3%)
No	329 (36.7%)
Median no. of acute signs/symptoms of COVID-19 (IQR) (*N* = 567)	4 (3–5)
Type of acute signs/symptoms of COVID-19^*b*^	
Cough	330 (36.8%)
Diarrhea	154 (17.2%)
Dyspnea	130 (14.5%)
Fever	276 (30.8%)
Headache	320 (35.7%)
Hypoxia (oxygen saturation ≤ 94% on room air)	58 (6.5%)
Malaise	363 (40.5%)
Nasal congestion	225 (25.1%)
Nausea/emesis	97 (10.8%)
Sore throat	363 (40.5%)
Median days from acute sign/symptom onset to test (IQR) (*N* = 567)	3 (5–8)

a COVID-19, coronavirus disease 2019; RT-PCR, reverse transcription PCR. Data are presented as *N* (%) unless otherwise specified.

b The sum of percentages does not equal 100% because participants could report any number of comorbidities and/or acute signs/symptoms of COVID-19.

**TABLE 2:** SARS-CoV-2 test results for participants providing same-day nasopharyngeal RT-PCR, nasopharyngeal rapid antigen, and saliva RT-PCR samples^*a*^

Result	Total (*N* = 896)
Nasopharyngeal RT-PCR	
Positive	269 (30.0%)
Negative	627 (70.0%)
*C_T_* value if positive, median (IQR) (*N* = 269)	25.7 (22.5–30.7)
Saliva RT-PCR (*N* = 894)	
Positive	242 (27.1%)
Negative	652 (72.9%)
*C_T_* value if positive, median (IQR) (*N* = 242)	29.0 (25.3–33.9)
Nasopharyngeal antigen	
Positive	201 (22.4%)
Negative	695 (77.6%)

a *C_T_*, cycle threshold; RT-PCR, reverse transcription PCR. Data are presented as *N* (%) unless otherwise specified.

**FIG 2:**
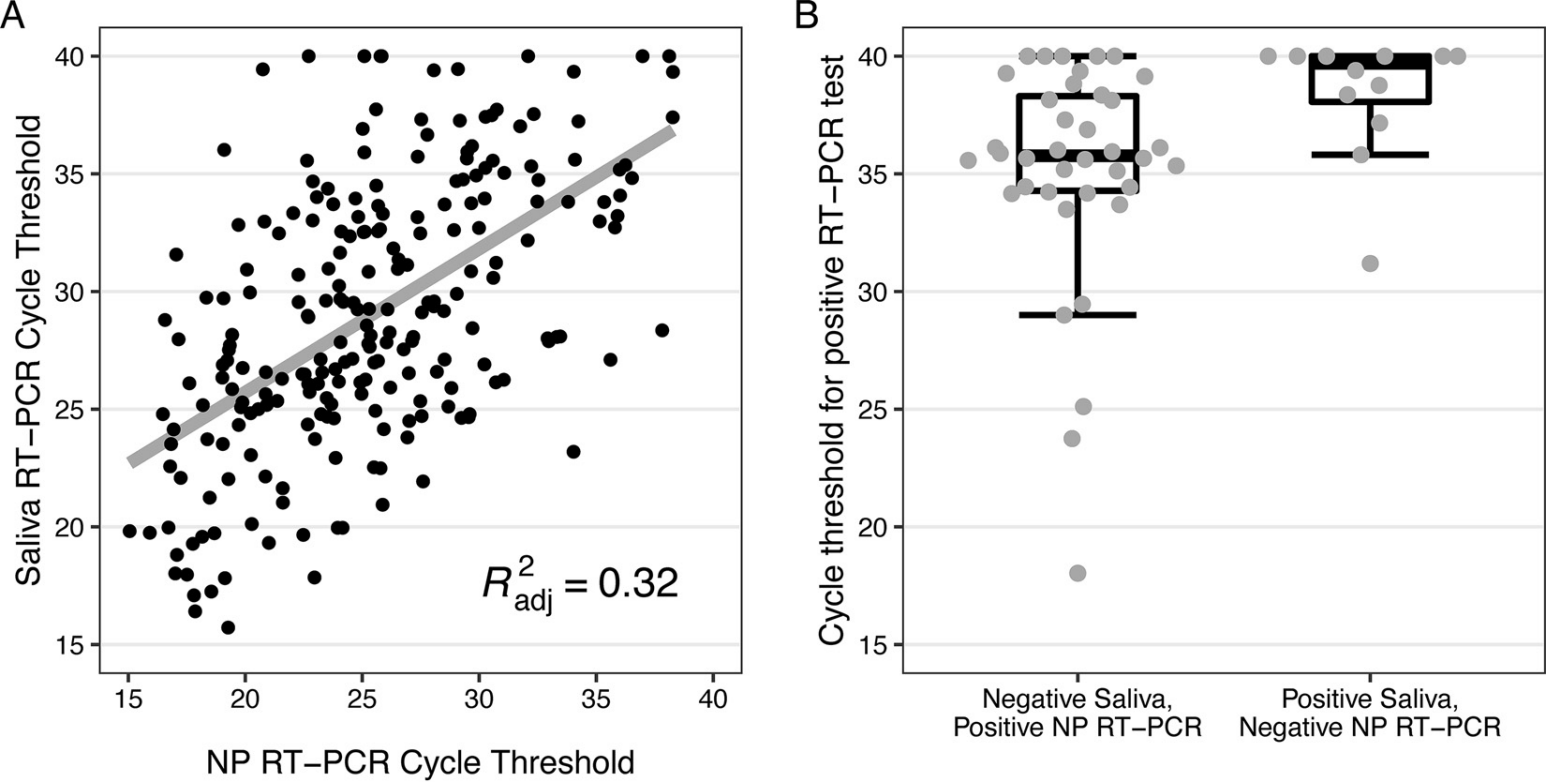
Saliva reverse transcription PCR (RT-PCR) cycle threshold by nasopharyngeal (NP) RT-PCR cycle threshold among participants with positive RT-PCR results on both same-day samples (panel A, *N* = 230). Positive RT-PCR results are defined as cycle thresholds of <45; the solid line shows a fitted linear regression with adjusted R^2^ shown on the plot. Box-and-whisker plot of cycle threshold by category of same-day saliva and nasopharyngeal RT-PCR samples with discrepant positive/negative results (panel B, *N* = 50). Lower and upper hinges correspond to the 25th and 75th percentiles, and the middle line corresponds to the median. Whiskers extend from the hinge to the smallest or largest value no further than 1.5 times the interquartile range (IQR) from the hinge.

The diagnostic performance of saliva RT-PCR and NP antigen for detection of SARS-CoV-2 is shown in Tables 3 and 4. Saliva RT-PCR showed 85.8% sensitivity (95% confidence interval [95% CI], 81.1% to 89.8%) and 98.1% specificity (95% CI, 96.7% to 99.0%) among all participants tested, with sensitivities of 87.3% (95% CI, 82.6% to 91.1%) and 37.5% (95% CI, 8.5% to 75.5%) among symptomatic and asymptomatic individuals, respectively. Notably, only 8/328 (2.4%) of asymptomatic individuals tested positive by NP RT-PCR, and only 3/8 (37.5%) of these tested positive by saliva RT-PCR. In this setting, the negative predictive value of saliva RT-PCR was 98.4%. The sensitivity of saliva RT-PCR was 97.4% (95% CI, 94.1% to 99.2%) among participants with *C_T_* values of ≤ 30 on NP RT-PCR. Overall, the observed sensitivities of saliva RT-PCR and the corresponding lower bounds of the 95% CIs exceeded the WHO-recommended threshold of 80% among (i) all participants, (ii) symptomatic participants, and (iii) participants with low *C_T_* values on NP RT-PCR. The observed specificities of saliva RT-PCR remained >95% regardless of symptom status and *C_T_* value on same-day NP RT-PCR. Figure 2A shows *C_T_* values for saliva RT-PCR versus nasopharyngeal RT-PCR among 230 participants with positive RT-PCR results on both samples (cycle thresholds < 45). Saliva samples showed a trend toward higher *C_T_* values than those obtained from NP swabs, with median *C_T_* values of 29.0 and 25.7, respectively (Table 2). Figure 2B shows a box-and-whisker plot of cycle thresholds among 50 same-day saliva and nasopharyngeal RT-PCR samples with discrepant positive and negative results; the median cycle threshold was >35 for samples with positive results for either (but not for both) saliva or NP swab samples.

**TABLE 3:** Performance characteristics of saliva RT-PCR for SARS-CoV-2 diagnosis, using same-day nasopharyngeal RT-PCR as the reference standard, by presence of acute signs/symptoms of COVID-19 and nasopharyngeal RT-PCR cycle threshold^*a*^

Saliva RT-PCR	Nasopharyngeal RT-PCR, *N* (%)			Sensitivity, % (95% CI)	Specificity, % (95% CI)	Predictive value, % (95% CI)	
	Positive	Negative	Total			PPV	NPV
Overall							
Positive	230 (85.8)	12 (1.9)	242	85.8 (81.1–89.8)	98.1 (96.7–99.0)	95.0 (91.5–97.4)	94.2 (92.1–95.8)
Negative	38 (14.2)	614 (98.1)	652				
Total	268	626	894				
Acute signs/symptoms of COVID-19^*b*^							
Positive	227 (87.3)	8 (2.6)	235	87.3 (82.6–91.1)	97.4 (94.9–98.9)	96.6 (93.4–98.5)	90.0 (86.3–93.0)
Negative	33 (12.7)	298 (97.4)	331				
Total	260	306	566				
No acute signs/symptoms of COVID-19^*b*^							
Positive	3 (37.5)	4 (1.2)	7	37.5 (8.5–75.5)	98.8 (96.8–99.7)	42.9 (9.9–81.6)	98.4 (96.4–99.5)
Negative	5 (62.5)	316 (98.8)	321				
Total	8	320	328				
*C_T_* value on nasopharyngeal RT- PCR of ≤30							
Positive	188 (97.4)	0 (0)	188	97.4 (94.1–99.2)	Not calculable	100.0 (98.1–100.0)	0.0 (0.0–52.2)
Negative	5 (2.6)	0 (0)	5				
Total	193	0	193				
*C_T_* value on nasopharyngeal RT- PCR of >30 to <45							
Positive	42 (56.0)	12 (1.9)	54	56.0 (44.1–67.5)	98.1 (96.7–99.0)	77.8 (64.4–88.0)	94.9 (92.9–96.5)
Negative	33 (44.0)	614 (98.1)	647				
Total	75	626	701				

a 95% CI, 95% confidence interval; COVID-19, coronavirus disease 2019; *C_T_*, cycle threshold; NPV, negative predictive value; PPV, positive predictive value; RT-PCR, reverse transcription PCR.

b Acute signs/symptoms of COVID-19 were defined as onset of any of the following within the 30 days before same-day sample collection: cough, diarrhea, dyspnea, fever, headache, hypoxia (oxygen saturation ≤ 94% on room air), malaise, nasal congestion, nausea/emesis, or sore throat.

**TABLE 4:** Performance characteristics of nasopharyngeal rapid antigen test for SARS-CoV-2 diagnosis, using same-day nasopharyngeal RT-PCR as the reference standard, by presence of acute signs/symptoms of COVID-19 and nasopharyngeal RT-PCR cycle threshold^*a*^

Nasopharyngeal rapid antigen test	Nasopharyngeal RT-PCR, *N* (%)			Sensitivity, % (95% CI)	Specificity, % (95% CI)	Predictive value, % (95% CI)	
	Positive	Negative	Total			PPV	NPV
Overall							
Positive	197 (73.2)	4 (0.6)	201	73.2 (67.5–78.4)	99.4 (98.4–99.8)	98.0 (95.0–99.5)	89.6 (87.1–91.8)
Negative	72 (26.8)	623 (99.4)	695				
Total	269	627	896				
Acute signs/symptoms of COVID-19^*b*^							
Positive	196 (75.1)	4 (1.3)	200	75.1 (69.4–80.2)	98.7 (96.7–99.6)	98.0 (95.0–99.5)	82.3 (78.0–86.1)
Negative	65 (24.9)	302 (98.7)	367				
Total	261	306	567				
No acute signs/symptoms of COVID-19^*b*^							
Positive	1 (12.5)	0 (0.0)	1	12.5 (0.3–52.7)	100.0 (98.9–100.0)	100.0 (2.5–100.0)	97.9 (95.7–99.1)
Negative	7 (87.5)	321 (100.0)	328				
Total	8	321	329				
*C_T_* value on nasopharyngeal RT- PCR of ≤30							
Positive	177 (91.2)	0 (0)	177	91.2 (86.3–94.8)	Not calculable	100.0 (97.9–100.0)	0.0 (0.0–19.5)
Negative	17 (8.8)	0 (0)	17				
Total	194	0	194				
*C_T_* value on nasopharyngeal RT- PCR of >30 to <45							
Positive	20 (26.7)	4 (0.6)	24	26.7 (17.1–38.1)	99.4 (98.4–99.8)	83.3 (62.6–95.3)	91.9 (89.6–93.8)
Negative	55 (73.3)	623 (99.4)	678				
Total	75	627	702				

a CI, confidence interval; COVID-19, coronavirus disease 2019; *C_T_*, cycle threshold; NPV, negative predictive value; PPV, positive predictive value; RT-PCR, reverse transcription PCR.

b Acute signs/symptoms of COVID-19 were defined as onset of any of the following within the 30 days before same-day sample collection: cough, diarrhea, dyspnea, fever, headache, hypoxia (oxygen saturation ≤ 94% on room air), malaise, nasal congestion, nausea/emesis, or sore throat.

NP antigen showed 73.2% sensitivity (95% CI, 67.5% to 78.4%) and 99.4% specificity (95% CI, 98.4% to 99.8%) among all participants tested, with sensitivities of 75.1% (95% CI, 69.4% to 80.2%) and 12.5% (95% CI, 0.3% to 52.7%) among symptomatic and asymptomatic individuals, respectively. Only 8/329 (2.4%) of asymptomatic individuals tested positive by NP RT-PCR, contributing to a negative predictive value of 97.9% for NP antigen. The sensitivity of NP antigen was highest, at 91.2% (95% CI, 86.3% to 94.8%), among participants with *C_T_* values of ≤30 on NP RT-PCR. Overall, the observed sensitivities of NP antigen and the corresponding lower bounds of the 95% CIs exceeded the WHO-recommended threshold of 80% only among participants with low *C_T_* values on NP RT-PCR. Observed specificities of NP antigen were >80% regardless of symptom status and *C_T_* value on same-day NP RT-PCR.

**FIG 3:**
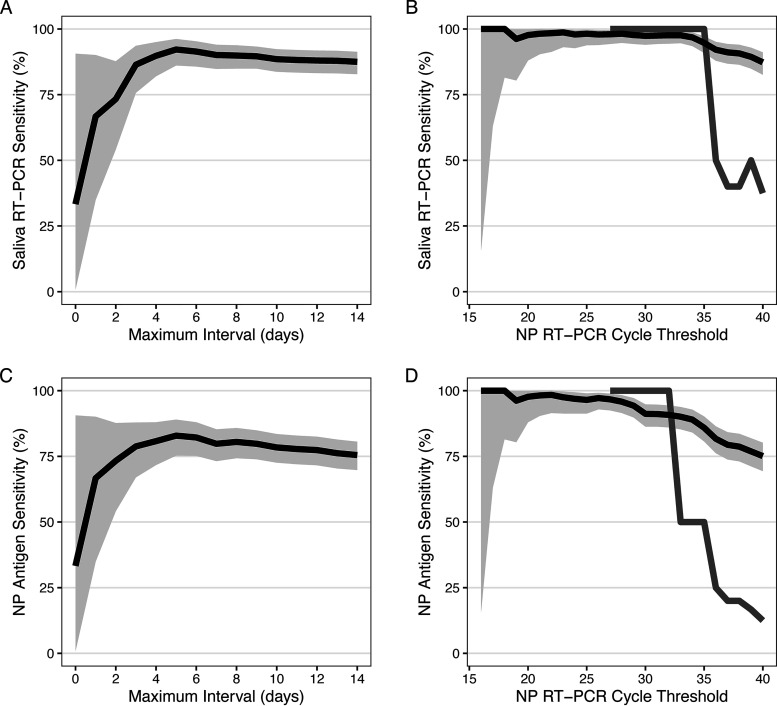
Sensitivity of saliva RT-PCR and nasopharyngeal rapid antigen test for SARS-CoV-2 diagnosis by (A and C) maximum interval from onset of acute signs/symptoms of COVID-19 to date of same-day specimen collection for nasopharyngeal RT-PCR, and by (B and D) cycle threshold on nasopharyngeal RT-PCR. Light gray areas show the exact binomial 95% confidence interval. Panels B and D show sensitivity among participants with and without acute signs/symptoms of COVID-19 (black and dark gray lines, respectively).

Figure 3 shows the sensitivity of saliva RT-PCR and NP antigen by the maximum interval from onset of acute signs/symptoms of COVID-19 to the date of same-day specimen collection for NP RT-PCR, and by cycle threshold on NP RT-PCR. Saliva RT-PCR showed a sensitivity of >80% among participants who underwent testing 3 to 14 days after symptom onset, and was most sensitive (>90% sensitivity) for those tested 5 to 7 days after symptom onset (Fig. 3A). In a sensitivity analysis restricted to 413 participants who presented within the first 7 days after symptom onset, the sensitivity of saliva RT-PCR was 90.1% (95% CI, 84.8% to 94.0%) (Table S1 in the supplemental material). Among all participants, samples with same-day NP RT-PCR *C_T_* values of <35 had >95% sensitivity on saliva RT-PCR (Fig. 3B).

The sensitivity of the NP antigen test exceeded 80% among participants who underwent testing 4 to 8 days after symptom onset (Fig. 3C). In a sensitivity analysis restricted to 450 participants who presented within the first 8 days after symptom onset, the sensitivity of NP antigen was 80.5% (95% CI, 74.3% to 85.8%) (Table S1). Among all participants, samples with same-day NP RT-PCR *C_T_* values of <37 and <33 had sensitivities of >80% and >90% on NP antigen, respectively (Fig. 3D).

## DISCUSSION

This community- and facility-based diagnostic validation study comprises a heterogenous urban population from Latin America and contributes a large pool of paired same-day samples from both symptomatic and asymptomatic participants from November 2020 to January 2021, when approximately 30% of the study population had SARS-CoV-2 infection according to the reference standard, NP RT-PCR. We found that saliva RT-PCR had acceptable sensitivity for the detection of SARS-CoV-2 among all-comers and symptomatic individuals, using the >80% sensitivity threshold recommended by WHO target product profiles for COVID-19 diagnostics. NP antigen did not achieve acceptable sensitivity except in those participants whose same-day NP RT-PCR result showed low *C_T_* values, consistent with a high viral load in the nasopharynx.

The performance of saliva RT-PCR in this study was similar to that reported in two recent meta-analyses, which reported pooled sensitivities of 83.2% (95% CI, 74.7% to 91.4%) and 85% (95% CI, 75% to 93%) across 16 and 23 studies, respectively (9, 13). In the meta-analyses, as in this study, saliva RT-PCR performance did not achieve parity with NP RT-PCR. Despite this, saliva samples provide several potential advantages, such as ease of sample collection and convenience for patients. In our sample, 12 same-day specimens from 4 asymptomatic and 8 symptomatic participants tested positive by saliva RT-PCR and negative by NP RT-PCR, all of which had saliva RT-PCR *C_T_* values of >30. Other studies have observed that saliva can be more sensitive than NP swabs for the detection of SARS-CoV-2 in certain scenarios, for example, among persons with previously confirmed SARS-CoV-2 infection (14), among asymptomatic individuals (15), and in samples collected from individuals 1 to 2 weeks after initial diagnosis (15, 16). Together, this body of evidence suggests that saliva RT-PCR can detect some cases of SARS-CoV-2 infection at low viral loads that are undetectable with NP RT-PCR.

This study shows that saliva RT-PCR performance is robust to different methods of RNA extraction. We followed the manufacturer’s instructions for the use of a locally available commercial RNA extraction kit, which differed from those for an RNA extraction kit used in a published reference protocol (17). The modified saliva processing method used less saliva and fewer reagents. These changes have the advantages of being easier for the individuals being tested and less demanding on the supply chain. They also did not appear to adversely impact the performance of the assay compared to those reported in the meta-analyses.

Among symptomatic participants, the sensitivity of the SD Biosensor STANDARD Q NP antigen test was lower than was reported in a recent Cochrane systematic review, which found 88.1% pooled sensitivity (95% CI, 84.2% to 91.1%) for this assay across 3 studies and 1,947 samples among 336 cases of SARS-CoV-2 infection (12). The systematic review deemed the STANDARD Q assay to be the only NP antigen test with pooled results of several studies that met the WHO acceptable criterion for sensitivity in symptomatic individuals. This assay was endorsed as a “replacement for laboratory-based RT-PCR when immediate decisions about patient care must be made, or where RT-PCR cannot be delivered in a timely manner.” In the Peruvian context, our finding that the overall NP antigen sensitivity in symptomatic participants was below the acceptable threshold of 80% compels us to make more modest recommendations regarding its routine use for patients presenting with acute signs/symptoms consistent with COVID-19. We found that NP antigen sensitivity exceeded 80% between 4 and 8 days after symptom onset among symptomatic participants, and among participants with high viral loads on same-day NP RT-PCR. Thus, in this population, we expect that NP antigen would be helpful for the diagnosis of SARS-CoV-2 infection among symptomatic patients several days after symptom onset or among those with severe presentations. These findings may be generalizable to populations outside Peru, given our heterogeneous sample and the fact that we performed NP antigen testing using the same procedures as those required for inclusion in the Cochrane systematic review. The sample size in this study was large relative to those of individual studies included in the Cochrane systematic review, and our results suggest that more caution is warranted when using the STANDARD Q NP antigen test under the conditions set forth by the systematic review’s endorsement. For asymptomatic participants, NP antigen sensitivity was particularly low, 12.5%, well below the pooled sensitivity of 69.2% (95% CI, 38.6% to 90.9%) reported in the systematic review (12). Despite its low sensitivity, the high specificity of NP antigen (100%) and low background prevalence of infection (2.4%) in our asymptomatic participant sample contributed to a high negative predictive value of 97.9%. Thus, when community transmission of SARS-CoV-2 is low, the STANDARD Q NP antigen test would be useful for mass screenings among asymptomatic participants, particularly with high testing frequencies at short time intervals as has been previously suggested (18).

This study has some limitations. We sought to validate these tests in a heterogenous sample. Consequently, our participant sample was derived from two distinct populations: (i) those being evaluated for inclusion in a seroprevalence study in the community and (ii) those seeking care in health facilities in North Lima. While this allowed us to secure large numbers of both symptomatic and asymptomatic participants, we recognize that the low prevalence of 2.4% NP RT-PCR positivity in the asymptomatic population resulted in wide confidence intervals for the sensitivity estimates for both saliva RT-PCR and NP antigen in this group. Another limitation is that, except for pulse oximetry, the timing and nature of acute signs/symptoms of COVID-19 were obtained through participant self-reporting and are thus subject to potential recall and desirability biases. We do note, however, that self-reported signs/symptoms were recorded before test results (and thus COVID-19 diagnoses) were known, which may have mitigated both potential biases. We also note that the timing of onset of self-reported acute signs/symptoms in our sample of symptomatic participants, at a median of 5 (IQR, 3 to 8) days before testing, played a direct role in the lower-than-predicted sensitivity estimates for the NP antigen test. Targeted use of NP antigen testing in certain populations, for example, those likely to have a high viral load, would improve test performance and approach the 91.2% sensitivity we detected among participants with high viral load by NP RT-PCR. Despite the emergence of new SARS-CoV-2 variants, the performance characteristics of saliva RT-PCR and NP antigen tests in diagnosing emerging variants, including Omicron, have shown to be comparable to their performance against ancestral SARS-CoV-2 (19–22). Our study provides a valuable contribution to the literature given its large sample size and the stratification of test results based on presence of symptoms, timing of symptoms from collection of samples, and corresponding *C_T_* values. In this era of rapid evolution in the field of COVID-19 diagnostics, validation of diagnostic tests will need continuous fine-tuning against newly emerging variants.

In summary, we showed that saliva RT-PCR achieved target performance for the detection of SARS-CoV-2. The increased patient convenience and health care worker protection achieved through self-collection of saliva may offer advantages which improve performance; for example, through early morning self-collection. The SD Biosensor STANDARD Q NP antigen test, however, only met the WHO-recommended threshold for sensitivity in participants mostly likely to be infectious (i.e., those with concomitant NP RT-PCR *C_T_* values of <30) (23) and in symptomatic individuals presenting 4 to 8 days after symptom onset. We observed a lower sensitivity of the STANDARD Q antigen test than has been previously reported in a meta-analysis. Confidence in the ability to rule out infection based on negative STANDARD Q NP antigen tests in large-scale screenings of asymptomatic individuals would require high testing frequency (18, 24).

## MATERIALS AND METHODS

### Ethics statement.

The study was approved by the ethics committee of the non-governmental organization Asociación Benéfica PRISMA. Written informed consent was obtained from all participants. Analysis was exempted from human subject research requirements by the Mass General Brigham Institutional Review Board (2021P000715).

### Study design, population, and setting.

From November 2020 to January 2021, we approached individuals living in communities and/or seeking care in health facilities in North Lima, Peru. Eligible individuals included both those with symptoms consistent with COVID-19, irrespective of known history of exposure, and those without symptoms who were known to have had recent exposure to persons diagnosed with COVID-19. We estimated a sample size of 375 symptomatic individuals, in whom 75 were expected to have a positive test for SARS-CoV-2 infection. Assuming that the observed point estimate for sensitivity would be 90% for either saliva RT-PCR or NP antigen, this sample size was selected to allow establishing, with 95% confidence, that the true sensitivity was ≥80% compared to that of NP RT-PCR. The 80% sensitivity threshold has been recommended in the WHO target product profiles for priority COVID-19 diagnostics (25). Recruitment of symptomatic and asymptomatic participants was planned until at least 75 positive RT-PCR tests had been reported among symptomatic participants.

### Samples.

Consenting participants provided saliva and two NP swab specimens. Three different methods were applied to samples from each participant: STANDARD Q COVID-19 Ag Test (SD Biosensor, Inc., Republic of Korea) performed on NP swab and COVID-19 genesig Real-Time PCR (Primerdesign Ltd., United Kingdom) performed on both saliva and NP swab specimens. Nasopharyngeal swabs were placed in universal transport medium (Beaver Biomedical Engineering Co. Ltd., China) and saliva was placed in an empty, sterile, 50-mL conical tube and maintained in cold chain during transfer to the laboratory for RNA extraction. Antigen tests and RT-PCR on NP swabs were performed according to manufacturers’ instructions (see the supplemental material).

### Viral RNA extraction.

In a biosafety level 3 (BSL-3) facility, 0.2 mL of each nasopharyngeal swab was treated by nucleic acid differential precipitation and lysis buffers from the CWBio Viral DNA/RNA kit (CoWin Biosciences, China) for later purification using silica columns. The samples were eluted in 60 μL of buffer.

Saliva samples were processed using the method described by Wyllie et al. (17, 26), with the following modifications: 0.2 mL saliva was used instead of 0.3 mL; saliva was treated with 10 μL proteinase K (10 mg/mL) instead of 20 μL (20 mg/mL); and RNA extraction was performed with the CWBio Viral DNA/RNA kit instead of the MagMAX Viral/Pathogen Nucleic Acid isolation kit (Thermo Fisher Scientific, Austin, Texas). The protocol modifications above were performed to accommodate the difference in RNA extraction kits.

### Data collection.

Participants self-reported demographic and clinical characteristics, possible exposure risks, and signs and symptoms of COVID-19 infection. Acute signs/symptoms potentially consistent with COVID-19 were defined as onset of any of the following within the 30 days before sample collection for NP RT-PCR: fever, malaise, headache, nasal congestion, sore throat, nausea/emesis, diarrhea, cough, dyspnea, or hypoxia (oxygen saturation of ≤94% on room air). All data were entered into a Microsoft SQL Server 2019 database (Microsoft Corporation, Redmond, Washington).

### Statistical analysis.

Analysis of saliva RT-PCR and NP antigen included all participants in whom all samples were collected on the date of the enrollment visit, during which standardized symptom assessment and pulse oximetry were performed. Point estimates and exact binomial confidence limits for sensitivity, specificity, and predictive values of saliva RT-PCR and NP antigen were calculated with NP RT-PCR as the reference standard, stratified by the presence of acute signs/symptoms of COVID-19 and NP RT-PCR *C_T_* values for positive tests (≤30 and >30 to <45). Participant characteristics, test results, and performance characteristics were presented as median and interquartile ranges for continuous variables and numbers and percentages for categorical variables. Analyses were performed using R version 4.1.0 (R Foundation for Statistical Computing, Vienna, Austria).
