# Supplementary material for: Diagnostic Performance Assessment of Saliva RT-PCR and Nasopharyngeal Antigen for the Detection of SARS-CoV-2 in Peru
Source: Microbiol Spectr. 2022 Jul 18;10(4):e00861-22. doi: 10.1128/spectrum.00861-22 (PMC9430815; doi:10.1128/spectrum.00861-22)
Supplement: Supplemental file 1 — Supplemental material. Download spectrum.00861-22-s0001.pdf, PDF file, 0.1 MB [file spectrum.00861-22-s0001.pdf]

Diagnostic Performance Assessment of Saliva RT-PCR and Nasopharyngeal Antigen for the Detection of SARS-CoV-2 in Peru

Roger I. Calderón,* Tulip A. Jhaveri,* Nadia N. Barreda, Oswaldo M. Sanabria, Marco A. Tovar, Jesús Peinado, J. Santiago Palomino, Claudio Ramirez, L. Fernando Llanos Zavalaga, Gissela Valderrama, Molly F. Franke, Carole D. Mitnick, Leonid Lecca,** Gustavo E. Velásquez**

* Co-first authors. These authors contributed equally to this manuscript, author order was determined on the basis of oversight of study implementation.
** Co-senior authors. These authors contributed equally to this manuscript, author order was determined on the basis of oversight of the data analysis and manuscript writing.

Sample collection, storage, and processing

One healthcare worker collected specimens for all paired tests: nasopharyngeal (NP) antigen, saliva reverse transcription polymerase chain reaction (RT-PCR), and NP RT-PCR.

<u>NP swab specimen collection</u>

The healthcare worker first collected an NP swab specimen and processed it with the SD Biosensor STANDARD™ Q antigen test for each participant. The NP antigen test result was read by one operator and reported within 15-30 minutes from sample collection. Following this, the healthcare worker collected a second NP swab specimen, from the ipsilateral nostril, for RT-PCR.

<u>Saliva specimen collection</u>

The healthcare worker advised participants to wait for at least 2 hours before providing a saliva specimen if they had eaten food. The healthcare worker then requested that the participant gently spit at least 3 to 5 mL of saliva into a sterile container for RT-PCR. Participants were asked to produce the saliva specimen within 5 minutes. The mean interval from time of sample collection to sample receipt in the lab was 260 minutes (SD 108 minutes).

<u>Specimen storage and processing</u>

NP swabs and saliva specimens collected for RT-PCR were kept in cold chain until they reached the Socios En Salud laboratory and thereafter were stored in the freezer at 2-8 degrees Celsius for NP swabs and -20 degrees Celsius for saliva. Processing of NP swabs was prioritized over saliva, and NP swabs were processed as soon as possible. On average, NP RT-PCR was reported on the same day the sample was collected, while the mean turnaround time from collection to reporting of saliva RT-PCR was 7.37 days.

Rapid SARS-CoV-2 antigen detection assay (NP antigen test)

The STANDARD™ Q COVID-19 antigen test (SD Biosensor, Inc., Suwon, Republic of Korea) is a rapid qualitative chromatographic immunoassay that detects specific SARS-CoV-2 antigen in respiratory specimens. Deltalab Peru E.I.R.L., an authorized representative of SD Biosensor, donated a portion of the NP antigen tests used in the study, product code Q-NCOV-01G / 09COV30D. This rapid antigen test device has two precoated lines on the result cassette section: control (C) and test (T) lines. The control region is coated with mouse monoclonal anti-chicken Igγ antibody; the test (T) region is coated with mouse monoclonal anti-SARS-CoV-2 antibody against SARS-CoV-2 antigen. Detectors for SARS-CoV-2 antigen presented in the specimen are mouse monoclonal anti-SARS-CoV-2 antibody conjugated with color particles. The antigen–antibody color particle complex migrates via capillary force and is captured by the mouse monoclonal anti-SARS-CoV-2 antibody coated on the test (T) region. The colored test (T) line's intensity depends on the amount of SARS-CoV-2 antigen present in the sample (1).

SARS-CoV-2 RNA detection using real-time RT-PCR

The COVID-19 genesig® Real-Time PCR test, (Primerdesign Ltd, Chandler's Ford, UK) targets the ORF1 ab gene of SARS-CoV-2 and was used for SARS-CoV-2 RNA detection according to the manufacturer's instructions. Briefly, 8 μL of extracted RNA was added to 12 μL of amplification mix to be used for amplification. The amplification conditions consisted of 1 cycle of 10 min at 55°C, 2 min at 95°C and followed by 45 cycles of 10 s at 95 °C, 60 s at 60°C. The results were analyzed in a LightCycler® 480 II Real Time Analyzer (Roche, Basel, Switzerland), a cycle threshold value < 45 for the target gene was defined as a positive result. In accordance with Primerdesign Ltd ISO 13485 certified Quality Management System, each batch of COVID-19 genesig® Real-Time PCR test kit was tested against predetermined specifications to ensure consistent product quality.

References

1. World Health Organization. 2020. WHO Emergency Use Assessment Coronavirus disease (COVID-19) IVDs. Public Report. Product: STANDARD Q COVID-19 Ag Test. EUL Number: EUL-0563-117-00. Outcome: Accepted. https://www.who.int/diagnostics_laboratory/eual/201019_final_pqpr_eul_0563_117_00_standard_q_covid19_ag_test.pdf. Retrieved 26 August 2021.

Supplemental Table 1. Performance characteristics of saliva RT-PCR and nasopharyngeal rapid antigen for SARS-CoV-2 diagnosis, using paired nasopharyngeal RT-PCR as the reference standard, by time of paired sample collection relative to onset of acute signs/symptoms of COVID-19.

	**Nasopharyngeal RT-PCR Reference Standard**						
**Test**	**Positive No. (%)**	**Negative No. (%)**	**Total No.**	**Sensitivity % (95% CI)**	**Specificity % (95% CI)**	**PPV % (95% CI)**	**NPV % (95% CI)**
**Saliva RT-PCR – sample collected within first 7 days of acute signs/symptoms of COVID-19***							
Positive	164 (90.1)	6 (2.6)	170	90.1 (84.8 – 94.0)	97.4 (94.4 – 99.0)	96.5 (92.5 – 98.7)	92.6 (88.5 – 95.6)
Negative	18 (9.9)	225 (97.4)	243				
Total	182	231	413				
**Saliva RT-PCR – sample collected after 7 days of acute signs/symptoms of COVID-19***							
Positive	63 (80.8)	2 (2.7)	65	80.8 (70.3 – 88.8)	97.3 (90.7 – 99.7)	96.9 (89.3 – 99.6)	83.0 (73.4 – 90.1)
Negative	15 (19.2)	73 (97.3)	66				
Total	78	75	153				
**Nasopharyngeal antigen – sample collected within first 8 days of acute signs/symptoms of COVID-19***							
Positive	161 (80.5)	3 (1.2)	164	80.5 (74.3 – 85.8)	98.8 (96.5 – 99.8)	98.2 (94.7 – 99.6)	86.4 (81.8 – 90.1)
Negative	39 (19.5)	247 (98.8)	286				
Total	200	250	450				
**Nasopharyngeal antigen – sample collected after 8 days of acute signs/symptoms of COVID-19***							
Positive	35 (57.4)	1 (1.8)	36	57.4 (44.1 – 70.0)	98.2 (90.4 – 100.0)	97.2 (85.5 – 99.9)	67.9 (56.6 – 77.8)
Negative	26 (42.6)	55 (98.2)	81				
Total	61	56	117				

CI = confidence interval; COVID-19 = coronavirus disease 2019; NPV = negative predictive value; PPV = positive predictive value; RT-PCR = reverse transcription polymerase chain reaction.
*Acute signs/symptoms of COVID-19 were defined as onset of any of the following within the 30 days before paired sample collection: cough, diarrhea, dyspnea, fever, headache, hypoxia (oxygen saturation ≤ 94% on room air), malaise, nasal congestion, nausea/emesis, or sore throat.
